# Effects of a parallel-arm randomized controlled weight loss pilot study on biological and psychosocial parameters of overweight and obese breast cancer survivors

**DOI:** 10.1186/s40814-017-0160-9

**Published:** 2017-07-10

**Authors:** Andrea Y. Arikawa, Beth C. Kaufman, Susan K. Raatz, Mindy S. Kurzer

**Affiliations:** 1 Department of Nutrition and Dietetics, University of North Florida, Jacksonville, FL - 1 UNF Drive, Jacksonville, FL 32224 USA; 2 Department of Food Science and Nutrition, University of Minnesota, St. Paul, MN - 1334 Eckles Ave, Saint Paul, MN 55108 USA; 3 Grand Forks Human Nutrition Research Center, USDA-ARS, Grand Forks, ND - 2420 2nd Avenue North, Grand Forks, ND 58206 USA

**Keywords:** Weight loss, Obesity, Diet, Breast cancer, Physical activity, Survivorship

## Abstract

**Background:**

Weight gain often occurs after breast cancer (BC) diagnosis and obesity along with sedentary behavior are associated with increased risk of BC recurrence and mortality. The primary objective of this study was to determine whether a significant weight loss, of approximately 10%, would lead to beneficial changes in biomarkers associated with cancer and/or cancer recurrence, and quality of life (QOL) in overweight and obese BC survivors.

**Methods:**

This parallel-arm study took place in Minneapolis, Minnesota, from January 2009 until March 2010. Participants were overweight and obese postmenopausal BC survivors who had completed treatment at least 3 months prior to enrollment and who did not smoke. Twenty-one BC survivors were randomized, via a random number generator computer software, to a 1000-calorie deficit feeding and exercise intervention (CR) or a weight management counseling intervention (WM) for 12 weeks followed by a 6-week follow-up. Body weight, biomarkers, and QOL were measured at baseline, weeks 6, 12, and 18. Body composition and fitness level were measured at only two time points.

**Results:**

Twenty-one women were enrolled into the study and 20 completed all time points. Weight loss occurred with both interventions. Body weight in CR changed from 85.5 (95% confidence interval (CI) 77, 94) kg to 76.7 (95% CI 68.1, 85.2) kg, whereas in WM it changed from 98.3 (95% CI 89.8, 106.8) kg to 93.2 (95% CI 84.6, 101.7) kg. Fitness in CR changed from 4.9 (95% CI 4, 5.8) to 6.3 (95% CI 5.4, 7.2). CR led to lower plasma levels of leptin, F2-isoprostanes, and CRP. Quality of life seemed to improve with both interventions, while sleep quality decreased only in CR.

**Conclusions:**

Overweight and obese BC survivors were able to adhere to a strict diet and exercise program, which significantly decreased body weight, increased fitness level, and improved biomarkers and QOL. However, the strict dietary intervention in CR seemed to decrease participants’ sleep quality and social relationships. Future larger randomized controlled trials should focus on behavioral modification and personalized nutrition counseling to help breast cancer survivors achieve a sustainable weight loss and fitness level.

**Trial registration:**

ClinicalTrials.gov identifier: NCT02940470.

## Background

In 2013, there were over 3 million breast cancer survivors in the USA, and with a 5-year relative survival rate of 89.7%, this number will continue to increase [1]. However, survivors often encounter physiological and psychological problems that may influence long-term prognosis, such as weight gain, decreased physical activity, and decreased quality of life (QOL). Research shows that 50–96% of breast cancer patients gain significant weight during and after therapy [2]. There is extensive evidence suggesting that being overweight or obese may increase risk of breast cancer recurrence, comorbidities, second cancers, and mortality [3]. It has been previously reported that obese breast cancer survivors exhibited anywhere from 30 to 540% increased risk of death [4].

Some suggested mechanisms involved in post-diagnosis weight gain are decreased physical activity (PA) as well as chemotherapy-related changes in metabolism [2, 5–8]. There is an association between PA after diagnosis and long-term prognosis, with decreases in recurrence risk of 50% in women who walked 3 to 5 h per week compared to inactive women [9] and significant decreases in mortality risk in survivors who walked 2 to 3 h per week [10]. PA combined with a dietary intervention designed to produce weight loss in overweight and obese survivors may reduce risk of disease recurrence, morbidity, and mortality [11].

There are several biological mechanisms underlying the relationship between obesity and breast cancer recurrence risk. Studies suggest that insulin, insulin-like growth factors, inflammatory status, adipokines, oxidative stress, vitamin D status, and estrogens may have a role in breast cancer recurrence and long-term prognosis [12]. Increased levels of fasting insulin and insulin-like growth factors have been associated with distant recurrence and death in breast cancer survivors [13]. C-reactive protein (CRP) is a marker of systemic inflammation and studies have linked increased pre-treatment CRP levels with decreased survival [14, 15] though this finding has not been consistently shown in other studies [16]. Leptin and adiponectin are adipokines that may influence recurrence risk [17]. Women with breast cancer may have dysregulation in several circadian systems, including hormonal rhythms [18] such as secretion of cortisol and melatonin. A lack of normal diurnal cortisol variation has been associated with early mortality in women with metastatic breast cancer [19] and low levels of melatonin have been associated with poor breast cancer prognosis as measured by tumor aggression [20], differentiation [21], and progression [22]. Elevated levels of F2-isoprostanes, a biomarker of whole-body oxidative stress [23], have been associated with increased risk of breast cancer among overweight and obese women [24]. However, it is not clear whether high levels of F2-isoprostanes are associated with increased risk of recurrence. Also of interest has been the relationship between Vitamin D status and breast cancer risk and progression. Adequate vitamin D status has been inversely associated with risk of breast cancer in prospective studies [25, 26], and a recent review has also reported a consistent inverse association between increased levels of vitamin D and decreased risk of progression and mortality of breast cancer [27]. Although more conclusive research is needed to establish a link between vitamin D levels and breast cancer prognosis, observational studies suggest that higher levels of vitamin D are associated with improved survival [28]. Most of the biomarkers mentioned above, with the exception of vitamin D, seem to be elevated in overweight and obese individuals. Thus, it would be expected that a diet and exercise intervention aimed at decreasing body weight would reduce the levels of these markers, which could decrease the risk of cancer recurrence.

Cancer diagnosis and treatment are also often associated with decreased quality of life (QOL) and may adversely affect psychosocial factors such as depression, anxiety, stress, self-esteem, psychological and emotional well-being, and fatigue that may last for long periods of time regardless of treatment outcome [29–32]. Physical activity and weight loss interventions after breast cancer diagnosis have mostly led to improved quality of life, reduced fatigue and depression, and improved sleep quality [33–35], although a recent clinical trial found an increase in depressive symptoms after weight loss [36].

Given the effects of obesity and a sedentary lifestyle on recurrence, comorbidities, quality of life, and mortality of breast cancer survivors, the purpose of this pilot study was to explore the effects of two weight loss interventions, a calorie restriction feeding plus exercise based weight loss intervention (CR) and a weight management counseling intervention (WM), on body composition, fitness, cancer-related biomarkers and quality of life among overweight and obese breast cancer survivors. When it comes to weight loss interventions, it is well established that those interventions aiming at behavioral change towards different lifestyle components are the most effective [37]. In breast cancer survivors, recent systematic reviews have shown similar findings [38, 39]. Our primary objective was to determine whether a significant weight loss, of approximately 10% over a short period of time, which was the target weight loss of CR, would lead to beneficial changes in the biological and psychosocial parameters described above. Given that most behavioral weight loss interventions do not typically achieve greater than 7% weight loss over a 6-month period, we designed a feeding intervention that would allow us to look at the effect of a greater weight loss over a short period of time.

## Methods

### Study design

We conducted a parallel-group randomized controlled trial where 21 overweight and obese breast cancer survivors were randomized, via a random number generator computer software, into one of two intervention groups: a calorie-restricted feeding plus exercise group (CR) or a weight management counseling group (WM). The rationale for choosing the two treatments was based on our primary objective. As we were interested in determining whether a significant weight loss of at least 10% would lead to significant changes in the biological and psychosocial parameters described above, it was not realistic to expect that a lifestyle behavioral modification would lead to that much weight loss over 12 weeks. Therefore, the CR group was designed to control the feeding of participants by providing all their meals for 12 weeks, as well as to include an exercise prescription to meet with a personal trainer twice per week during these 12 weeks. The weight management counseling intervention was designed as the comparison group because it was based on behavioral and lifestyle modification, which is considered the standard of practice.

Participants were studied for a total of 18 weeks, consisting of 12 weeks of active intervention plus a 6-week follow-up period. Body weight was measured at baseline; weeks 6, 12, and 18 and body composition was evaluated at baseline and week 12. The American College of Sports Medicine (ACSM) recommends a minimum of 15–20 weeks is needed to measure efficacy of a fitness program [40], thus fitness level was measured at baseline and week 18. Due to the nature of the interventions described, participants and study staff were not blinded to treatment allocations. The only blinding that took place was that of study staff conducting laboratory analysis of biological parameters.

### Study participants

Overweight and obese breast cancer survivors were recruited in Minneapolis, Minnesota for this pilot study between January and August 2009 via emails sent to breast cancer survivors affiliated to the Love/Avon Army of Women (www.armyofwomen.org) and via advertisements posted at the Fairview University Medical Center and Breast Center. Interested women contacted study coordinators through provided phone or email information on recruitment materials. If women were determined eligible after an initial screening interview, a screening clinic visit for verification of eligibility status was scheduled. Inclusion criteria included: postmenopausal, defined as experiencing at least 12 months without a menstrual period, diagnosed with operable invasive breast cancer (TNM staging system T1-3, pN0-pN3, M0) and treated with mastectomy or with lumpectomy and radiation, completed all surgery, radiation, and systemic chemotherapy at least 3 months prior to enrollment, BMI ≥ 27 kg/m^2^, less than seven servings of alcohol/week, willing to be randomized into either group, not planning to move away from the area during the period of the study, and non-smoker. Exclusion criteria included serious illness requiring medical treatment, inability to participate in physical activity due to severe disability, history of schizophrenia, psychosis or untreated major depression, unwilling to commute to study site once/wk, and failure to provide written informed consent. This study was approved by the University of Minnesota Institutional Review Board. Written informed consent was obtained from all participants prior to beginning any study activities. (ClinicalTrials.gov Identifier: NCT02940470).

### Intervention

Women were randomized via a random number generator computer software to either a weight management counseling group (WM) or a 1000-calorie deficit feeding plus exercise group (CR). Prior to randomization, a registered dietitian assessed each participant’s current dietary intake and physical activity level in order to determine total energy expenditure, which was estimated as the calculated basal energy expenditure adjusted for reported physical activity and dietary intake. An energy prescription of 1000-kcal deficit from estimated total energy expenditure was given to each woman.

### Weight management counseling group (WM)

Women randomized into WM participated in weekly 1-h weight management classes supervised by a registered dietitian for 12 weeks. Individualized guidelines for the energy-restricted diet, menu plans, and recommendations for increased physical activity levels were prescribed during the first class. The remaining classes covered topics related to short-term and long-term weight loss, including exercise and behavior modification.

### Calorie restricted diet plus exercise group (CR)

Women randomized into the diet plus exercise group were provided all meals, freshly prepared, for 12 weeks. Meals were picked up daily Monday–Friday, with the Friday pick-up including meals for the weekend. The meals included breakfast, lunch, dinner, and a snack reflecting a deficit of 600–900 kcal per day (which when combined with estimated energy expenditure via the exercise program, equaled the 1000 kcal deficit per day). Macronutrient breakdown per day was as follows 55% of total energy from carbohydrates, 15% from protein, and 30% from fat, with 125–150 mg cholesterol per 1000 kcal, 12–15 g dietary fiber per 1000 kcal and providing 100% of the reference dietary intake for all essential vitamins and minerals.

Women randomized into CR were also given a progressive exercise intervention, which combined both aerobic and strength training, and included a membership to a fitness center. The exercise program was supervised by a certified trainer, who met with each participant twice per week for the first 4 weeks, and once per week thereafter. Exercise prescription was based on ACSM guidelines for weight loss and prevention of weight gain in overweight and obese adults [41]. Recommendations specific for cancer survivors have since been published [42] and our exercise intervention fit within these recommendations. The energy expended during exercise varied between 100–400 kcal per day in order to obtain a total energy deficit of 1000 kcal per day when combined with the dietary caloric deficit. Initially, participants exercised 3 days per week for 15–20 min each time at 60–70% of calculated heart rate maximum (220 − age). The frequency of exercise gradually increased throughout the period of the intervention until participants were exercising five to six times per week, for a total of 150–225 min per week. Progressive weight training sessions took place twice per week. A recent study found that in survivors with lymphedema, slowly progressive weight lifting had no significant effect on limb swelling and resulted in a decreased incidence of exacerbations of lymphedema, reduced symptoms, and increased strength [43]. The weight training sessions were constantly monitored to ensure compliance and to make sure participants maintained their form. Participants were asked to wear heart rate monitors during their aerobic exercise sessions and keep logs of their aerobic and strength training workouts throughout the study.

### Limited contact intervention

After the first 12 weeks of the intervention, women in both groups were followed for an additional 6 weeks. Women in the CR group were instructed to continue both aerobic and weight lifting exercise at the recommended levels of 150–225 min per week of aerobic activity, and twice weekly weight lifting sessions. They also continued to keep a log of their exercise. Women in the WM were instructed to use the knowledge and skills they learned during the previous 12 weeks towards continuing weight management or further weight loss. Also, women in both groups were given printed materials containing information on a healthy diet for cancer survivors and a sample weekly menu to provide guidance if desired.

### Anthropometrics and body composition

Body weight was assessed at baseline, weeks 6, 12, and 18 to the nearest 0.1 kg, using an electronic scale (Scale Tronix, White Plains, NY). Women in CR also weighed themselves each day they picked up their food (up to 5 days/week) and recorded weight in provided diet compliance logs. Height was measured at baseline without shoes to the nearest 0.1 cm (Scale Tronix, White Plains, NY). Body mass index (BMI) was calculated as the weight in kilograms divided by height in square meters (kg/m^2^). Body composition was assessed at baseline and week 12 using dual energy x-ray absorptiometry (DXA) (Lunar Prodigy, GE Medi, Madison, WI).

### Fitness assessment

A sub-maximal treadmill test was performed at baseline and week 18. After a 5-min warm-up, participants walked on the treadmill at a steady speed (2.0–3.0 miles per hour depending on participant ability), and percent grade on the treadmill was increased by 2% every 2 min until the participants reach 80% of their age-predicted maximum heart rate (max HR), defined as 220 − age. Heart rate was measured using Polar Heart Rate monitors (Polar Electro Inc., Woodbury, NY). This workload was converted into metabolic equivalents (MET) using a standard conversion formula [44]. The test was performed by a certified exercise physiologist who was blinded to group assignment. This assessment was also used as a symptom-limited exercise stress test in order to detect any potential adverse effects of the intervention.

### Sample collection

Blood and urine samples were obtained at baseline, and weeks 6, 12, and 18. Participants were asked to collect all of their urine output for 24 h separated into day time (7 am–10 pm) and night time (10 pm–7 am) urine, for measurement of cortisol and 6-sulfatoxymelatonin. Insulin, glucose, insulin-like growth factor (IGF)-1, IGF binding protein (IGFBP-3), F2-isoprostanes, 25-hydroxyvitamin D (25(OH)D), and inflammatory markers were measured in plasma or serum at all four time points, with the exception of F2-isoprostanes, which were measured at baseline and weeks 0, 12, and 18 and 25(OH)D, which was measured at baseline and week 12.

### Laboratory measurements

Free F_2_-isoprostanes were measured in plasma by a gas chromatography-mass spectrometry (GC-MS)-based method [45] at the Molecular Epidemiology and Biomarker Research Laboratory (MEBRL), University of Minnesota, Minneapolis, Minnesota. Fasting blood glucose and plasma insulin levels were measured at the Fairview University Diagnostic Laboratories (Minneapolis, MN). Glucose levels were assessed using colorimetric reflectance spectrophotometry. Insulin levels were assessed by chemiluminescent immunoassay (Immulite, Diagnostic Products Corporation, Los Angeles, CA). The measure of insulin resistance was determined by the homeostatic model assessment (HOMA) index. The HOMA index was calculated by multiplying fasting plasma insulin (mmols/L) by fasting glucose (mmols/L) and then dividing by 22.5. HOMA correlates well (*r* = −0.83) with insulin sensitivity as measured by the gold standard euglycemic clamp. We also calculated insulin sensitivity using the quantitative insulin sensitivity check index (QUICKI = 1/[log_(fasting insulin)_ + log_(fasting glucose)_].

Commercially available Enzyme-Linked Immunosorbent Assays (ELISAs) were used to measure levels of IGF-1, IGFBP-3, leptin, adiponectin, C-reactive protein (CRP), urinary cortisol (in 24-h urine), interleukin-6 (IL-6), and 6-sulfatoxymelatonin (in overnight urine), a major metabolite of melatonin in urine. All analytes were assayed using kits purchased from R&D Systems Inc (Minneapolis, MN) with the exception of 6-sulfatoxymelatonin which was assayed by kit purchased from ALPCO Diagnostics (Salem, NH). Serum 25(OH)D was assayed at Heartland Assays (Ames, IA) by competitive chemiluminescence immunoassay (CLIA) using the DiaSorin LIAISON 25-OH Vitamin D Total assay [46, 47]. Intra- and interassay coefficients of variation were 6.6 and 2.8% for CRP, 8.2 and 6.1% for IL-6, 4.2 and 5.1% for adiponectin, 4/1 and 4.7% for leptin, 2.4 and 3.6% for IGF-1, 3.3 and 3.7% for IGFBP-3, 7.8 and 7.4% for 6-sulfatoxymelatonin, 3.3 and 2.3% for urinary cortisol, and 8.1 and 11.2% for 25(OH)D.

### Quality of life

Quality of life (QOL) will be assessed at baseline and weeks 6, 12, and 18 using the World Health Organization’s quality of life assessment instrument WHOQOL-BREF [48], which is a 26-item version of the WHOQOL-100 [49].

### Sample size

Due to the pilot nature of this study, a sample size calculation was not performed. The initial aim of the study was to recruit between 20 and 25 participants because having 10 to 12 participants per group seemed like a sufficient sample size to inform our group of trends in the levels of the biomarkers of interest as a consequence of weight loss.

### Statistical methods

Descriptive statistics were generated by cross-tabulation for categorical variables and by means for continuous variables. In order to compare the treatment groups at the four time points, analysis of variance with repeated measures was performed. Data for all continuous variables are expressed as mean (95% confidence interval).

## Results

**Fig. 1:**
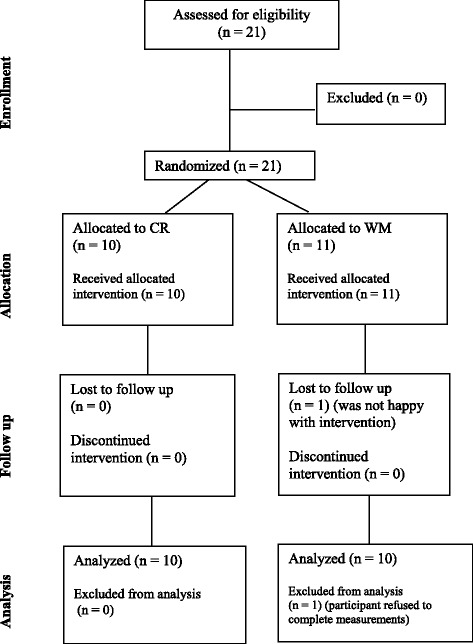
Flowchart of participants in the study. CR = 1000-calorie deficit feeding and exercise intervention group; WM = weight management counseling group

We recruited a total of 21 women for the study, and 20 women successfully completed all study procedures (Fig. 1). One participant dropped out of WM because her expectations were not met by the intervention. Baseline characteristics (means, standard deviations, and frequencies) of all participants are shown in Table 1.

**Table 1:** Baseline characteristics of study participants (*n* = 10 per group)

	CR (*n* = 10)	WM (*n* = 10)
Age (years)	54.7 ± 8.4	58.4 ± 7.6
Weight (kg)^a^	85.5 ± 8.3	98.3 ± 19
BMI (kg/m^2^)^a^	31.5 ± 3.3	36.9 ± 7.7
Body fat (%)	49.4 ± 4.8	50.6 ± 3.7
Bone mineral density (g/cm^2^)	1.18 ± 0.11	1.26 ± 0.09
Fitness level (METs)	4.89 ± 1.48	4.80 ± 0.97
Glucose (mg/dL)^b^	80.7 ± 9.8	96.5 ± 21.8
Insulin (mU/L)^b^	9.0 ± 4.1	19.4 ± 20.7
25(OH)vitamin D (ng/mL)	35.0 ± 9.4	33.1 ± 12
Use of vitamin D supplements		
Yes	4	3
No	6	7
Stage of breast cancer		
Stage I	2	4
Stage II	7	4
Stage III	1	2
Chemotherapy/radiotherapy		
Yes	10	9
No	0	1
Endocrine therapy		
Aromatase inhibitors	4	5
Selective estrogen receptor modulators	4	3
None	2	2
Mastectomy		
Yes	5	6
No	3	0
Lumpectomy	2	4
Race		
White	10	10
Other	0	0

Values presented as mean ± SD, or frequencies

*Abbreviations*: *CR* Calorie restricted diet plus exercise group, *WM* weight management counseling group

Every woman randomized into CR performed at least one weight lifting session per week with 40% adhering to the twice weekly weight lifting sessions for the entire 18 weeks. There was a high adherence rate for aerobic exercise training, with 70% of these women exercising for 150 (range 78–240) minutes per week. There was 100% compliance to the weekday food pick-ups, with no missed meals. In WM, adherence was 77.5% and it was calculated by dividing the number of sessions attended by all women by the total number of sessions given.

One participant in the WM was diabetic (baseline glucose and insulin levels were 146 mg/dL and 77 mU/L, respectively) and another participant in the same treatment group had impaired glucose tolerance (glucose and insulin levels were 116 mg/dL and 18 mU/L, respectively).

Table 2 shows body weight (kg), total body fat mass (TBFM, kg), total body fat percent, and total body lean mass (kg) by treatment group. There was improvement in these parameters in both groups, although the average weight loss in the WM group was largely driven by one participant who lost 12.9 kg (9.5% of her body weight).

**Table 2:** Body weights and body composition of breast cancer survivors (*n* = 10 per group)

	Week 0	Week 6	Week 12	Week 18
Body weight (kg)				
CR	85.5 (77, 94)	80.3 (71.8, 88.8)	77.5 (69, 86)	76.7 (68.1, 85.2)
WM	98.3 (89.8, 106.8)	95.7 (87.2, 104.2)	94.1 (85.6, 102.6)	93.2 (84.6, 101.7)
Body fat (%)				
CR	49.6 (46.3, 53)	–	45.2 (41.9, 48.5)	–
WM	50.4 (47, 53.7)	–	49.6 (46.3, 53)	–
Body fat (kg)				
CR	42.1 (35.7, 48.5)	–	35.4 (29, 41.8)	–
WM	46.1 (39.7, 52.5)	–	43.6 (37.2, 49.9)	–
Lean mass (kg)				
CR	42 (37.9, 46)	–	41.6 (37.5, 45.7)	–
WM	45.2 (41.1, 49.3)	–	43.9 (39.9, 48)	–

Values displayed as mean (95% confidence interval)

*Abbreviations*: *CR* calorie restricted diet plus exercise group, *WM* weight management counseling group

Women in CR showed an improvement in fitness level at week 18 (6.3 mets (95% CI: 5.4, 7.2) compared to baseline 4.9 mets (95% CI: 4, 5.8). This improvement in fitness level was not as evident in the WM group whose initial and week 18 fitness levels were 4.8 mets (95% CI: 3.9, 5.7) and 5.4 mets (95% CI: 4.5, 6.3), respectively.

Table 3 shows levels of glucose, insulin, insulin resistance, and IGF proteins over the four time points of the study. Table 4 shows levels of markers of inflammation, stress, and other hormones associated with breast cancer risk over time. Leptin, CRP and F2-isoprostanes showed improvement over time in the CR group.

**Table 3:** Levels of glucose, insulin and insulin related proteins in breast cancer survivors over time (*n* = 10 per group)

	Week 0	Week 6	Week 12	Week 18
Glucose (mg/dL)				
CR	80.7 (72.1, 89.3)	79.8 (71.2, 88.4)	75.5 (66.9, 84.1)	78.7 (70.1, 87.3)
WM	96.5 (87.9, 105.1)	91.7 (93.1, 100.3)	92.4 (83.8, 101)	94.5 (85.9, 103.1)
Insulin (mU/L)				
CR	9 (2.7, 15.3)	7.2 (0.8, 13.5)	6 (0, 12.3)	6.9 (0.6, 13.2)
WM	19.4 (13.1, 25.7)	14.7 (8.4, 21)	13.6 (7.3, 19.9)	13.5 (7.2, 19.8)
HOMA index				
CR	1.8 (-0.3, 3.9)	1.4 (0, 3.5)	1.1 (0, 3.3)	1.4 (0, 3.5)
WM	5.4 (3.3, 7.6)	3.6 (1.5, 5.7)	3.2 (1.1, 5.4)	3.3 (1.2, 5.4)
QUICKI				
CR	0.6 (0.6, 0.7)	0.7 (0.6, 0.8)	0.8 (0.7, 0.9)	0.8 (0.7, 0.9)
WM	0.5 (0.4, 0.6)	0.6 (0.5, 0.7)	0.6 (0.5, 0.7)	0.6 (0.5, 0.7)
IGF-1 (ng/mL)				
CR	103.8 (80, 127.6)	106.8 (83, 130.6)	108 (84.3, 131.8)	117.4 (93.7, 141.2)
WM	99.3 (75.5, 123.1)	85 (61.3, 108.8)	98 (74.3, 121.8)	105.1 (81.4, 128.9)
IGFBP-3 (ng/mL)				
CR	1855 (1476, 2234)	1833 (1455, 2212)	1792 (1413, 2171)	1740 (1362, 2119)
WM	1900 (1521, 2279)	1578 (1199, 1957)	1812 (1434, 2191)	1827 (1448, 2205)

Values displayed as mean (95% confidence interval)

*Abbreviations*: *CR* calorie restricted diet plus exercise group, *WM* weight management counseling group, *HOMA* homeostasis model assessment, *QUICKI* quantitative insulin sensitivity check index, *IGF-1* insulin-like growth factor-1, *IGFBP-3* insulin-like growth factor binding protein-3

**Table 4:** Levels of markers of inflammation, stress, and other hormones associated with breast cancer risk in breast cancer survivors over time (*n* = 10 per group)

	Week 0	Week 6	Week 12	Week 18
Leptin (ng/mL)				
CR	47 (34.8, 59.3)	21.5 (9.3, 33.8)	19 (6.7, 31.2)	26.2 (14, 38.5)
WM	53.8 (41.5, 66)	37.3 (25, 49.5)	36.8 (24.5, 49)	38.2 (26, 50.5)
Adiponectin (μg/mL)				
CR	12.8 (9.3, 16.2)	9.6 (6.2, 13)	11.1 (7.7, 14.5)	13.8 (10.4, 17.2)
WM	7.7 (4.3, 11.1)	6.9 (3.5, 10.3)	7.9 (4.5, 11.3)	8.3 (4.9, 11.7)
CRP (mg/L)				
CR	6 (2.1, 9.8)	4.3 (0.4, 8.1)	3.2 (0, 7.1)	3.8 (0, 7.7)
WM	5.2 (1.3, 9)	4.4 (0.6, 8.2)	7.4 (3.6, 11.3)	5.9 (2, 9.7)
Interleukin-6 (pg/mL)				
CR	2.7 (1.8, 3.6)	2 (1.1, 2.9)	2.2 (1.3, 3.1)	2.4 (1.6, 3.3)
WM	2.8 (1.9, 3.7)	2.4 (1.5, 3.3)	3.3 (2.4, 4.2)	2.7 (1.8, 3.6)
F2-isoprostanes (ng/mL)				
CR	71.5 (52.5, 90.4)	–	50 (31.1, 68.9)	52.9 (33.9, 71.8)
WM	81 (62, 100)	–	62 (43.1, 81)	64.9 (46, 83.9)
Cortisol (ng/mL)				
CR	26.4 (18.3, 34.6)	21.2 (13.1, 29.4)	27.1 (19, 35.3)	23.1 (14.9, 31.3)
WM	40.3 (32.1, 48.5)	28.3 (20.1, 36.5)	36.1 (27.9, 44.3)	37 (28.8, 45.1)
6-Sulfatoxymelatonin (ng/mg cr)				
CR	8.1 (3, 13.3)	8.6 (3.5, 13.8)	5.8 (0.1, 11.6)	10 (4.9, 15.2)
WM	15.8 (10.7, 21)	13.1 (7.7, 18.5)	11.6 (6.4, 16.7)	16.9 (11.8, 22.1)

Values displayed as mean (95% confidence interval)

*Abbreviations*: *CR* calorie restricted diet plus exercise group, *WM* weight management counseling group, *CRP* C-reactive protein

Levels of 25(OH)D were measured at weeks 0 and 12 only. At week 0, levels of 25(OH)D were 32.9 (95% CI: 25.5, 40.3) and 35.2 (95% CI: 28.1, 42.2) in the WM and CR groups, respectively. At week 12, levels of 25(OH)D were 33.1 (95% CI: 26.1, 40.2) and 41.4 (95% CI: 34.4, 48.4) in the WM and CR groups, respectively.

Table 5 shows the overall quality of life (QOL) and specific QOL parameters over the four time points of the study.

**Table 5:** Quality of life and sleep scores of breast cancer survivors over time (*n* = 10 per group)

	Week 0	Week 6	Week 12	Week 18
Quality of life				
CR	71.2 (66.1, 76.4)	74.2 (69, 79.3)	75.8 (70.8, 81)	77 (71.6, 82.4)
WM	59 (53.6, 64.4)	62.6 (57.2, 68)	60.4 (54.7, 66.2)	62.3 (54.7, 66.2)
Physical health				
CR	57.7 (50.9, 64.5)	61.4 (54.6, 68.2)	64 (57.2, 70.8)	66.1 (58.9, 73.3)
WM	48.1 (40.9, 55.3)	55.1 (47.9, 62.3)	54 (46.4, 61.6)	50 (42.4, 57.6)
Psychological				
CR	60.7 (53.5, 67.8)	68.9 (61.7, 76)	72.5 (65.3, 79.6)	71.6 (64, 79.1)
WM	50.9 (43.3, 58.4)	54.2 (46.7, 61.8)	52.5 (44.5, 60.5)	53.2 (45.3, 61.2)
Social relationships				
CR	78.8 (68.8, 88.8)	80.6 (70.6, 90.6)	78.7 (68.7, 88.7)	79.2 (68.7, 89.7)
WM	62.4 (51.9, 72.9)	63.3 (52.8, 73.8)	59.4 (48.2, 70.5)	69.5 (58.3. 80.6)
Environment				
CR	87.7 (80.6, 94.8)	85.8 (78.7, 92.9)	88.2 (81.1, 95.3)	91 (83.5, 98.4)
WM	74.6 (67.1, 82)	77.8 (70.3, 85.2)	75.9 (68, 83.8)	76.6 (68.7, 84.5)
Sleep quality				
CR	7.3 (5, 9.6)	6 (3.7, 8.3)	5.1 (2.8, 7.4)	4.4 (2, 6.9)
WM	7.7 (5.2, 10.1)	7.3 (4.9, 9.8)	7.5 (4.9, 10.1)	7.7 (5.1, 10.4)

Values displayed as mean (95% confidence interval)

*Abbreviations*: *CR* calorie restricted diet plus exercise group, *WM* weight management counseling group

## Discussion

The primary objective of this randomized pilot trial was to determine whether a significant weight loss of approximately 10% would favorably change biological and psychosocial measures in breast cancer survivors. Our study examined the effects of a calorie restriction plus exercise feeding intervention (CR) compared to a weight management counseling group (WM) on weight, body composition, fitness, cancer-related biomarkers, and quality of life in overweight and obese breast cancer survivors. While several randomized controlled trials examining the effects of weight loss interventions on health outcomes of breast cancer survivors have been published and a few more are currently being conducted, many of these trials focused on change in weight as the primary outcome, and few have examined the impact of weight loss on psychosocial measures [39]. Overweight or obese status, along with having a sedentary lifestyle, is thought to increase breast cancer risk and recurrence. Women in both groups successfully lost weight and percent body fat (%BF) during the trial. However, women in CR lost more weight and %BF compared to WM at weeks 6, 12, and 18. CR also had a significantly greater increase in fitness compared to baseline and also compared to WM. Both groups were able to maintain or continue to reduce body weight during the limited contact follow-up (weeks 12–18), although the CR participants showed greater weight loss and greater improvements in fitness level at week 18. To our knowledge, only one other randomized controlled trial of a weight loss intervention in this population resulted in a weight loss of 10% [50]. It has been suggested that this weight loss level normalizes several metabolic parameters that are adversely affected by obesity and is therefore the recommended percent to lose by the National Institutes of Health to reduce risk of metabolic and cardiovascular diseases [51]. Williamson and colleagues found that weight loss of ≥9.1 kg in overweight women resulted in a 25% reduction in all cause, cardiovascular, and cancer mortality [52]. We observed an average loss of 9.6 kg in CR, which accounted for 11% of initial body weight. In addition, while other studies of weight loss reported a significant loss of lean mass, our study demonstrated that it is feasible to have substantial weight loss (≥10%) without concomitant loss of lean mass. Incorporating physical activity, and specifically weight training, into a weight loss program is key to maintaining or increasing muscle mass. Along with reduced adiposity and maintained lean mass, we found a significant 29.4% increase in fitness in CR. Research has shown that breast cancer survivors who engage in one or more hours/week of moderate physical activity have a lower risk of cancer recurrence ranging from 20 to 50%[10, 53]. In our study, 100% of the women reached a minimum of 1 h/week, 60% of the women averaged between 2–3 h/week, and 30% of the women averaged over 3 h/week.

There have also been few published studies to date looking at the effects of weight loss on a comprehensive number of biological markers such as the ones described here. Several randomized controlled trials to date have measured insulin and glucose, but few have measured adipokines or inflammatory markers other than CRP [54]. Our study indicates that levels of IGF-1 may increase with weight loss, an expected finding as BMI has been inversely associated with IGF-1 levels [55]. Levels of IGFBP-3 seemed to be lower as a consequence of weight loss. Studies have shown that high levels of IGFBP-3 predicted distant recurrence of breast cancer in postmenopausal women [56] and high levels of IGFBP-3 have been found in tissue of breast tumors associated with poor prognosis [57].

Obesity is consistently associated with vitamin D deficiency in adults [58, 59]. When it comes to the relationship between 25(OH)D levels and breast cancer risk, data from case-control studies indicate a statistically significant inverse relationship, whereas data from prospective studies do not support such relationship [27]. Recent meta-analyses have reported statistically significant relationship between higher levels of 25(OH)D and improved breast cancer prognosis [60–62]. Levels of 25(OH)D increased in the CR group and this finding suggests that weight loss may beneficially affect vitamin D status in breast cancer survivors.

F2-isoprostanes are considered one of the best biomarkers of systemic in vivo oxidative stress [63]. Systemic oxidative stress may play a role in progression of breast cancer. Breast cancer treatment may involve the generation of free radicals which induce oxidative stress and kill cancer cells; however, high levels of free radicals in the body may also adversely impact breast cancer prognosis and a recent case-control study looking at urinary levels of F2-isoprostanes in deceased breast cancer patients versus surviving patients found a significant inverse association between urinary levels of F2-isoprostanes and mortality [64]. To our knowledge, this is the first study to assess levels of F2-isoprostanes in breast cancer survivors participating in a weight loss intervention. Weight loss resulted in decreases in plasma F2-isoprostanes in both groups. Based on findings from exercise intervention studies, it seems that exercise alone also decreases levels of F2-isoprostanes even in the absence of weight loss [65, 66], suggesting that interventions combining calorie restriction with exercise training may be the most effective to reduce F2-isoprostanes in obese individuals. Cortisol levels are often used as a surrogate of stress and previous studies have found disrupted circadian rhythms of diurnal cortisol in breast cancer patients [19, 67]. There were no clear changes in 24-h cortisol levels in this pilot study, and given the findings from previous studies, diurnal, rather than 24-h cortisol levels, may be considered in a future trial.

Similar to other studies of physical activity and weight loss interventions in survivors [38], there were slight improvements in overall quality of life (QOL) in the CR group. However, this group also showed a decrease in sleep quality and social relationships over the study period. The CR intervention was very intense and involved a strict low-calorie diet regime, which may have been a reason why sleep quality and social relationships (the women may have reduced social activities in order to avoid food temptations, and since all meals were provided for them, they may have decreased activities that involved dining out) decreased.

## Conclusions

Some of the strengths of our study include the randomized controlled study design, the inclusion of a limited contact follow-up period, the comprehensive array of variables measured, particularly F2-isoprostanes, which have not been measured in any previous trials, and the significant weight loss achieved in the CR group. Regarding the comprehensive array of variables measured, the rationale for assessing all these biomarkers was the reported associations between obesity or energy balance and changes in all these variables. Limitations of this study were an imbalance between the two groups with respect to baseline body weight, a highly-tailored feeding intervention, and the lack of a dietary assessment measurement to monitor dietary adherence in the WM group. Also, since we combined physical activity with a diet intervention, we cannot separate their individual effects, and we cannot determine the separate effects of the weight training and aerobic exercise. The authors acknowledge that a highly-tailored feeding intervention as the one described here does not provide breast cancer survivors with tools and resources needed to succeed in their weight management goals. However, the primary objective of this pilot trial was to determine whether a significant weight loss led to changes in biological and psychosocial parameters over a short period of time. Indeed, we found that a 10% decrease in body weight and a 16% decrease in fat mass were accompanied by improvements in several biomarkers that may translate into lower recurrence, morbidity, and mortality risks. Our next step will be to develop an intervention that combines elements of the CR group with elements of the WM group resulting in behavioral modification and personalized nutrition counseling to maximize weight loss and help breast cancer survivors maintain body weight in the long term. Future larger randomized controlled trials are needed to determine how to sustain long-term weight loss and fitness and how these can significantly affect prognosis of breast cancer and mortality from a psychosocial perspective as well as a biological perspective, particularly considering systemic biomarkers such as plasma F2-isoprostanes, C-reactive protein, insulin, IGF-1, IGFBP-3, and 25(OH)-vitamin D. It is also important to determine whether there is an optimal level of weight loss and exercise level that should be sustained over time to reduce recurrence and improve prognosis in breast cancer survivors.

## Abbreviations

%BF: Percent body fat
BC: Breast cancer
CR: Calorie-restricted diet and exercise intervention
CRP: C-reactive protein
HOMA: Homeostatic model assessment (measure of insulin resistance)
IGF-1: Insulin-like growth factor 1
IGFBP-3: Insulin-like growth factor binding protein 3
IL-6: Interleukin 6
METs: Metabolic equivalents
PA: Physical activity
QOL: Quality of life
TBFM: Total body fat mass
TBLM: Total body lean mass
WM: Weight management counseling intervention
